# Density-Dependent Recycling Promotes the Long-Term Survival of Bacterial Populations during Periods of Starvation

**DOI:** 10.1128/mBio.02336-16

**Published:** 2017-02-07

**Authors:** Sotaro Takano, Bogna J. Pawlowska, Ivana Gudelj, Tetsuya Yomo, Saburo Tsuru

**Affiliations:** a Department of Bioinformatic Engineering, Graduate School of information Science and Technology Osaka University, Osaka, Japan; b Biosciences, University of Exeter, Exeter, United Kingdom; c Graduate School of Frontier Biosciences, Osaka University, Osaka, Japan; d Exploratory Research for Advanced Technology (ERATO), Japan Science and Technology Agency (JST), Osaka, Japan; Emory University

## Abstract

The amount of natural resources in the Earth’s environment is in flux, which can trigger catastrophic collapses of ecosystems. How populations survive under nutrient-poor conditions is a central question in ecology. Curiously, some bacteria persist for a long time in nutrient-poor environments. Although this survival may be accomplished through cell death and the recycling of dead cells, the importance of these processes and the mechanisms underlying the survival of the populations have not been quantitated. Here, we use microbial laboratory experiments and mathematical models to demonstrate that death and recycling are essential activities for the maintenance of cell survival. We also show that the behavior of the survivors is governed by population density feedback, wherein growth is limited not only by the available resources but also by the population density. The numerical simulations suggest that population density-dependent recycling could be an advantageous behavior under starvation conditions.

## IMPORTANCE

How organisms survive after exhaustion of resources is a central question in ecology. Starving *Escherichia coli* constitute a model system to understand survival mechanisms during long-term starvation. Although death and the recycling of dead cells might play a key role in the maintenance of long-term survival, their mechanisms and importance have not been quantitated. Here, we verified the significance of social recycling of dead cells for long-term survival. We also show that the survivors restrained their recycling and did not use all available nutrients released from dead cells, which may be advantageous under starvation conditions. These results indicate that not only the utilization of dead cells but also restrained recycling coordinate the effective utilization of limited resources for long-term survival under starvation.

## INTRODUCTION

Microorganisms comprise much of Earth’s biodiversity and occupy virtually every niche, subjecting themselves to a wide range of environmental pressures, such as nutrient exhaustion. Indeed, a great number of bacteria are known to live under extreme nutrient limitation (1). How microbes survive in extreme or nutrient-poor environments is one of the central questions in ecology.

In laboratory culture, long-term survival during starvation was also observed in the bacterium *Escherichia coli* (2–4). After the majority of *E. coli* cells died (death phase), a small proportion of the cells remained viable for months (long-term stationary phase) (2–5). What enabled survival during starvation? Previous studies showed the emergence of mutants within a population that possessed growth advantages under long-term starvation; some of these mutants could utilize nutrients from dead cells, which enhanced their ability to grow using amino acids as a carbon source (6–9). Thus, it is plausible that one novel mechanism for survival under starvation conditions is the use of nutrients derived from dead cells (6).

Although there have been numerous reports explaining long-term survival by focusing on specific mutants, using a molecular genetic approach, the importance and mechanism of recycling activity in long-term survival are yet to be verified at the population level.

First, the social behaviors observed in many organisms are usually population density dependent (10, 11), but density dependency of long-term survival of *E. coli* cells in starvation has not been demonstrated. If *E. coli* cells need to perform recycling (i.e., the growth of cells using nutrients released from dying cells) to survive starvation, the number of dead cells would change the viability of the population during starvation. Thus, the initial population density would determine the viability during long-term starvation. A previous study observed the survival kinetics of starved *E. coli* cells starting from various initial cell densities; however, this study focused only on the survival kinetics at the beginning of starvation and not on the recycling activity (12).

The mechanism underlying how death and recycling enable populations of cells to survive for a long period has not been studied quantitatively. One rationale is that the mechanism that maintains the viability of the cells at a constant level during long-term stationary phase is the balancing of growth and death rates (4, 13). However, how cell growth and death are controlled to maintain the viability of the cells at a constant level has not been explored in detail. The mechanism and conditions that are sufficient to stop the decrease in the survivors during long-term stationary phase have been verified by neither experimental nor mathematical approaches.

In this study, we conducted ecological laboratory experiments using *E. coli* cells under starvation conditions in combination with mathematical models. We used this system to show how *E. coli* bacteria maintained a population of viable cells at a constant level through recycling activity, by quantitatively estimating the death and growth rates during starvation. Our analysis of the viability of *E. coli* cells during starvation shows that the survival of the population is primarily governed by the environment constructed by the cells themselves after undergoing the death phase. Moreover, the initial population density affects the survival rate during the long-term stationary phase. We also show that specific mutations may not be essential for survival during starvation, and therefore, survival under starvation conditions may be considered an ecological rather than an evolutionary process. Further analysis shows that not only the balancing of active growth and death but also the attenuation of death and recycling can maintain viability during the long-term stationary phase. *E. coli* cells can restrain their recycling activity and, thus, do not use all of the available nutrients. Interestingly, this behavior is highly dependent on the population density of the viable cells. These lines of evidence were rationally combined together in a mathematical model without contradictions with each other and contributed to the explanation showing that the recycling activity was sufficient to maintain life under starvation conditions.

## RESULTS

### Population density-dependent cell survival rate during starvation.

To determine whether the initial population density was important for cell survival, we measured the survival kinetics of glucose-starved *E. coli* cells for 30 days, starting from various initial cell densities. To reduce the influence of the evolutionary mechanisms, we used strain MDS42, which has a lower mutation rate than its original strain, MG1655. In previous studies, the initially added nutrients were not completely eliminated from the cultures (2–4, 14). Therefore, there is a possibility that the cells require the residual nutrients remaining in the culture after exponential growth for their survival, instead of using the nutrients released from the dead cells. Here, we exchanged the medium for M63 minimal medium without glucose after the cells had grown to a density of 10^9^ cells/ml, to eliminate all nutrients remaining in the culture (referred to as 10^9^-cell/ml starved culture below).

**FIG 1:**
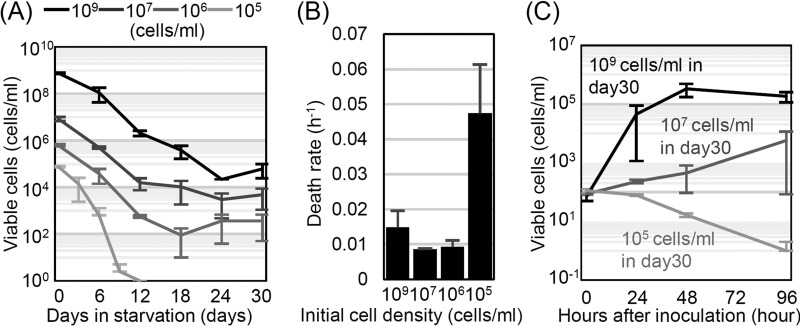
The survival kinetics of *E. coli* cells in glucose-depleted culture is dependent on initial cell density. (A) Numbers of viable cells in starvation cultures with various initial population densities (*n* = 3). (B) Population death rates at the beginning of starvation (days 0 to 6) estimated for starving populations with various initial population densities (*n* = 3). (C) Numbers of viable cells in cultures composed of freshly prepared 100-cell/ml cultures inoculated into supernatants from various day 30 starved cultures started with the cell densities indicated in panel A (*n* = 2). The estimation of the number of viable cells is consistent with the results shown in panel A. In all panels, the error bars indicate the standard deviations.

Figure 1A shows the temporal survival kinetics of the viable cells under glucose starvation conditions as estimated by CFU counts. At the beginning of starvation, the population was in the death phase, where the number of viable cells decreased in all samples. When the cells entered starvation at densities of 10^9^, 10^7^, and 10^6^ cells/ml, they entered into long-term stationary phase, where their viability stopped decreasing when the number of viable cells reached each positive threshold. However, when the cells entered starvation at a density of 10^5^ cells/ml, their viability decreased more rapidly (Fig. 1B, death rate) (analysis of variance [ANOVA], *n* = 3, *F*_3,8_ = 4.07, *P* = 0.0020) and immediately dropped below 1 cell/ml.

Next, we measured the growth kinetics of freshly prepared *E. coli* cell cultures in the supernatants from starved cultures with various initial cell densities. We hypothesized that the apparently different behaviors of cultures with 10^9^- and 10^5^-cell/ml densities was due to differences in the concentrations of substrates released from the dead cells during starvation.

Figure 1C shows the growth kinetics of the newly prepared fresh cell cultures in each of the supernatants. The growth of the fresh cell cultures changed significantly depending on the supernatants into which they were inoculated (cell concentrations at day 2, ANOVA, *n* = 2, *F*_2,3_ = 9.55, *P* = 0.00071; cell concentrations at day 4, ANOVA, *n* = 2, *F*_2,3_ = 6.94, *P* = 0.045). In the supernatants from the cultures with initial densities of 10^9^ and 10^7^ starving cells per ml, the freshly prepared cells grew to densities of ~10^5^ and 10^4^ cells/ml, which was comparable to the number of viable cells in stationary phase (Fig. 1A). Conversely, the freshly prepared cells did not grow in the supernatant obtained from the culture with an initial density of 10^5^ cells/ml.

Based on these results, it is clear that the initial population density of the starving cells affected cell survival by changing the concentrations of substrates in the supernatant. Thus, the residual nutrients in the culture were not necessary for long-term survival. The more cells that starve, the higher the concentration of substrates that becomes available for cell survival. This finding supports the hypothesis that the maintenance of population survival during starvation is governed by cell death and the nutrients released by the cells, as previously suggested (4, 6, 8). This result also suggests that maintaining a high cell density during exponential growth or while forming aggregates (e.g., biofilms) is important for the enhanced ability of *E. coli* cells to survive starvation.

It should be noted that sufficient resources remain for another 10^5^ cells/ml to grow in the day 30 supernatant of a starved culture initiated at 10^9^ cells/ml (Fig. 1C) in which 10^5^ cells/ml had already survived (Fig. 1A). This would suggest that the survivors in long-term stationary-phase cultures did not utilize all of the nutrients available in the cultures and that the remaining nutrients could allow the same number of cells as was in the 10^9^-cell/ml starved culture at day 30 to grow again. We investigated this possibility, and the results are presented in the next section.

We can also conclude that adaptive mutations may not be necessary for survival during long-term starvation. Freshly prepared homogeneous populations at low densities grew and survived in the environment where 10^9^ and 10^7^ cells/ml were cultured, and the final number of viable cells was nearly the same as that in the long-term stationary phase (days 24 and 30 in Fig. 1A) of the original starving *E. coli* populations initiated at densities of 10^9^ and 10^7^ cells/ml. There is little possibility of mutations arising and diversification of genotypes in the freshly prepared populations, because the mutation rate of the strain used in this study is ~9.6 × 10^−4^ (substitution/replication × genome). Additionally, the growth of freshly prepared cells was not drastically inhibited even when they were cultured with cells that had undergone starvation for 30 days (see Fig. S1 in the supplemental material). This suggests that survivors in the long-term stationary phase do not have a significant advantage for growth using nutrients released from the dead cells compared to fresh cells in this time frame. Therefore, probably the nutrients or molecules released from the dead cells, rather than changes in the cell genotype, determined cell behavior during long-term starvation. For details of our reasoning, see Text S1 in the supplemental material.

10.1128/mBio.02336-16.1 FIG S1  Competition experiments between cells starved for 30 days and freshly prepared cells. (A) Design of the experiments. To test whether evolution or selection during 30 days’ starvation enhances the ability of viable cells to grow using nutrients from dead cells, we diluted day 30 MDS42 strain cell cultures (10^9^ cells/ml) with supernatants from the same cultures and mixed the diluted cultures with freshly prepared cell cultures of strain MDS42*ΔgalK*::*gfp-*Km^r^. (B) Growth curves of starved and fresh cells in the mixed population in the supernatant were estimated by CFU. To estimate the viability of both strains, cell cultures were plated on M63 agar plates with or without kanamycin at 40 μg/ml. The viability of fresh cells was estimated from a viable count on M63 agar with kanamycin, and that of starved cells was estimated by subtracting the viable count on M63 agar with kanamycin from that on M63 agar without kanamycin. (C) Transition of population ratios in mixed cultures from day 0 to 4. No significant changes were detected in this time course (ANOVA, *n* = 3, *F*_3,8_ = 4.07, *P* = 0.29). The error bars indicate the standard deviations. Download FIG S1, PDF file, 0.3 MB. Copyright © 2017 Takano et al. 2017 Takano et al. This content is distributed under the terms of the Creative Commons Attribution 4.0 International license.

10.1128/mBio.02336-16.2 TEXT S1  Rationale for the survival in long-term stationary phase without *de novo* mutations and the model description and analysis for bacterial survival under starvation conditions using three variables. Download TEXT S1, PDF file, 0.3 MB. Copyright © 2017 Takano et al. 2017 Takano et al. This content is distributed under the terms of the Creative Commons Attribution 4.0 International license.

### Excessive supply of nutrients from the cells during starvation.

How bacterial cells stop dying and maintain their viability at a constant level is unclear. We hypothesized that a population could transit from the death phase to the long-term stationary phase when the accumulation of substrates from the dead cells in the environment was sufficiently high to sustain a small minority of viable cells.

**FIG 2:**
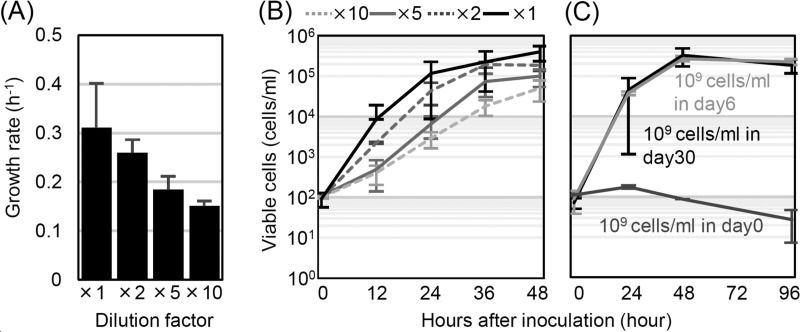
Cell growth rates dependent on the substrates and growth curves in supernatants from day 6 and day 30 starved cultures. (A) To verify the effect of the substrates in the supernatants, we inoculated newly prepared cells into supernatants from 10^9^-cell/ml starved cultures at day 30 with different dilution factors. The growth rate of each culture was estimated by curve fitting the data from 0 to 48 h to a logistic function (*n* = 3) (see Materials and Methods). (B) Growth kinetics in the supernatants (*n* = 3) whose growth rates are shown in panel A. (C) Growth curves of newly prepared cells in supernatants from cultures with different starvation times. Amounts of 100 freshly prepared cells per ml were inoculated into the supernatants from cultures with the indicated starvation times and starved cultures. The estimation of the number of viable cells is consistent with the results shown in Fig. 1. The error bars indicate the standard deviations of duplicate experiments.

To test this hypothesis, we determined how the substrate concentrations affected the population growth rates of freshly prepared cell cultures at a low cell density. Figure 2A and B show the growth rates and kinetics in supernatants diluted with M63 medium without glucose. We obtained the supernatants by centrifuging and filtering starved cultures that had an initial density of 10^9^ cells/ml. Dilution of the supernatants significantly changed the growth rate of the freshly prepared cells (Fig. 2A, growth rate) (ANOVA, *n* = 3, *F*_3,8_ = 4.07, *P* = 0.035). The more diluted the supernatant, the more slowly the cells grew. Therefore, the population growth rate changes depending on the substrates contained in the supernatant, and we can describe this rate as a function of the substrates, similar to the Monod equation (15).

Next, we tested whether the resource concentration alone could drive the transition between the death and long-term stationary phases. To this end, we studied the cells’ behavior in the supernatants of cultures obtained at entry into starvation (day 0), death phase (day 6), and stationary phase (day 30). If our hypothesis regarding the transition from the death to the stationary phase is correct, the final number of viable cells should be different in each supernatant. Figure 2C shows the cell growth curves in each supernatant. The supernatant from the day 0 culture could not support the growth of freshly prepared cells (Fig. 2C), indicating that the substrates are not sufficient for cellular growth in the culture at the beginning of starvation. That is, residual nutrients or nutrients leaked during centrifugation or washing out of the culture are not sufficient for cell growth or survival in the starvation culture. Surprisingly, the freshly prepared cells grew to a density of up to 10^5^ cells/ml in the supernatants from both the day 6 and day 30 cultures (Fig. 2C), and the cell concentrations of these two samples did not differ significantly after 4 days (*P* = 0.239, one-tailed *t* test). This result demonstrated that the substrates in the death phase (day 6) probably reached a concentration that was sufficient to maintain a small population of viable cells until the stationary phase (day 30).

These results suggest that the concentrations of substrates accumulating in the culture can alter bacterial growth. However, the substrate concentration is not the sole factor that determines the transition from the death to the long-term stationary phase. The cells keep dying in the death phase even though there are plenty of substrates to support growth and stop death, which is similar to the long-term stationary phase.

### Population density-dependent growth in starved cultures.

The concentration of the substrates after undergoing the death phase was sufficient to maintain cell viability, but the population growth rate changed from the death phase at the beginning of starvation to the long-term stationary phase. How do the cells change their growth and/or death rates between the death and long-term stationary phases? One possibility is that cell growth and/or death is controlled by the population density of the viable cells. Bacterial growth arrest may occur, depending on the number of viable cells, through the secretion of certain small molecules (e.g., colicin) (16) or by cell-to-cell contact (17).

**FIG 3:**
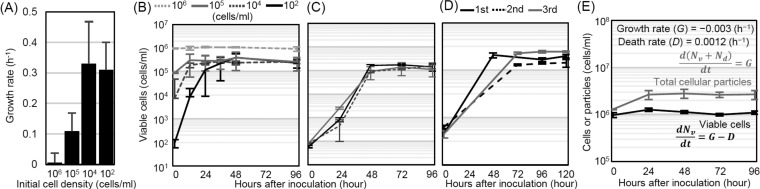
Population density-dependent cell growth in supernatants. (A) To test the effect of the number of viable cells on the growth rate, we inoculated freshly prepared cell cultures at various densities into the supernatants from 10^9^-cell/ml starved cultures at day 30. The growth rates were estimated by fitting the data obtained from 0 to 96 h to a logistic equation (see Materials and Methods) (*n* = 3). (B) Growth kinetics in the supernatants of cultures started with various initial cell densities (*n* = 3). (C) To test whether residual substrates remained in the culture, 100 freshly prepared cells per ml were inoculated into supernatants from the 96-h-incubated cultures whose viable-cell counts are shown in panel B (*n* = 3). (D) To test whether cells that had stopped growing at ~10^5^ cells/ml could grow again in the supernatant after the first growth cessation (first round), the first-round cultures were diluted 1,000-fold with supernatant obtained from the first-round cultures at 120 h after inoculation (second round). This procedure was performed again (third round) (*n* = 2) (for details, see Fig. S3 in the supplemental material). (E) Temporal dynamics of total cellular particles and the numbers of viable cells in long-term stationary-phase cultures estimated by flow cytometry. The sums of the viable cells and the dead cells were estimated by counting total cellular particles (gray line). The numbers of viable cells were estimated by counting CFU (black line). The data shown are the average results from triplicate experiments. The indicated growth and death rates are estimated from the slope of each line from the 24-h to 96-h experiments (for details, see Materials and Methods). In all experiments, the estimation of the number of viable cells was consistent with the results shown in Fig. 1. The error bars indicate the standard deviations.

Thus, we hypothesized that the viable-cell density could affect cell population growth during starvation. We verified this hypothesis by estimating the population growth rate of a culture starting at a low initial cell density in the supernatant obtained from a culture incubated from 10^9^ cells/ml under starvation conditions for 30 days. Figure 3A and B show the population growth rates and kinetics for various initial cell densities. We found that the higher the initial population density, the lower the population growth rate of that population (Fig. 3A, growth rate) (ANOVA, *n* = 3, *F*_3,8_ = 4.35, *P* = 0.044). As the population density of the viable cells approached a threshold (~10^5^ cells/ml), the cells stopped growing. Thus, the cells changed their population growth rate depending on the density of the viable cells.

If the bacteria stopped growing due to the exhaustion of nutrients in the culture, we would expect little opportunity for regrowth in the supernatant obtained after the cells reach a plateau (i.e., 96 h after the inoculation of the cells in the experiment whose results are shown in Fig. 3B). Figure 3C shows the growth kinetics of freshly prepared cell cultures in the supernatants obtained at 96 h from the experiment whose results are shown in Fig. 3B. Surprisingly, the cells regrew to a density of ~10^5^ cells/ml in the supernatant in which other cells stopped growing at a density of ~10^5^ cells/ml. No significant differences in cell concentration were observed between the original starved cultures at day 30, fresh cell cultures grown in the supernatant (first round, Fig. 3B), and those of the second round (Fig. 3C; see also Fig. S2 in the supplemental material). Although there was a decrease (not significant) in the final cell concentration from the first to the second round of growth in the supernatants, the final cell concentrations in the second-round cultures did not change dramatically compared to the number of viable cells in the original culture (10^9^-cell/ml starved culture at day 30) (Fig. 1A; see also Fig. S2). This indicates that the 10^5^ surviving cells per ml in the original starved cultures left nutrients sufficient to allow subsequent growth of more than 10^5^ cells/ml again. This also supports the interpretation that cells do not stop growing because nutrients are exhausted in long-term stationary-phase culture. However, it is possible that freshly prepared cell cultures carry over nutrients that have been exhausted in the supernatant, allowing the fresh cells to regrow to a density of ~10^5^ cells/ml. We investigated this possibility and confirmed that supplying nutrients from freshly prepared cell cultures is not necessary for the cells’ regrowth. One hundred cells per milliliter that had already grown to ~10^5^ cells/ml regrew to a density of over 10^5^ cells/ml (Fig. 3D). Regrowth to ~10^5^ cells/ml was observed at least three times following serial transfer in the supernatant without any supplementation of nutrients or fresh cell cultures (Fig. 3D). These results clearly show that exhaustion of the substrates is not the primary cause of the cessation of growth. The results in Fig. 3D also show that the final cell concentration in the supernatant did not gradually decrease with serial transfer. In both the experiment whose results are shown in Fig. 3D and the experiment whose results are shown in Fig. S2, there was a decrease in the final cell concentration from the 1st to the 2nd round of growth in the supernatant. Thus, it is possible that nutrients in the supernatant gradually decreased as the cells grew, resulting in final cell concentrations in the supernatant that decreased as serial transfer continued. However, the final cell concentration in the 3rd-round culture in the experiment whose results are shown in Fig. 3D was higher than that in the 1st-round culture. Nutrients sufficient for the growth of over 4 × 10^5^ cells/ml (final cell concentration of the 1st-round cultures) remained even after the 2nd round of growth. Thus, nutrients sufficient for the growth of 4 × 10^5^ cells/ml remained for at least three cycles (Fig. 3D). This also supports the view that the cells stopped growing before nutrients sufficient to grow the same number of cells again were exhausted.

10.1128/mBio.02336-16.3 FIG S2  Growth cessation before exhaustion of nutrients in a supernatant. Average viable cell concentrations in the original 10^9^-cell/ml starved cultures at day 30 (*n* = 8) (the experimental condition is consistent with the results shown in Fig. 1A) and in populations regrown in the supernatants at 96 h after inoculation (first round [*n* = 3] and second round [*n* = 6]) (as shown in Fig. 3B and C). The experimental design is as presented in Fig. 1A and 3B and C and their legends. To clarify differences among the samples, we plotted the results against a linear scale. The error bars indicate the standard deviations. Download FIG S2, PDF file, 0.1 MB. Copyright © 2017 Takano et al. 2017 Takano et al. This content is distributed under the terms of the Creative Commons Attribution 4.0 International license.

From the results described above, we found that the cells stopped growing above a certain threshold, with the remaining nutrients sufficient to grow the same number of cells again in the supernatant from long-term stationary-phase culture, and the cells that had slowed down their growth could restart fast growth when the number of viable cells decreased. Therefore, we conclude that the cells can control their population growth depending on the density of the viable cells and propose that cells are able to sense their density and respond to the concentration of the substrate per capita.

It should be noted that growth arrest with sufficient substrates remaining for cells to regrow to the same density in the environment was not observed for a stationary-phase culture in a standard minimal medium with glucose instead of the supernatant of the long-term stationary-phase culture (see Fig. S4 in the supplemental material). Initially, there were sufficient nutrients for growth to 10^8^ to 10^9^ cells/ml, but cells regrew to a density of no more than 10^4^ to 10^5^ cells/ml in the supernatant of the stationary-phase culture (see Fig. S4A, 24 h). Therefore, growth arrest before the exhaustion of nutrients by population density feedback, which was not observed in glucose-supplemented minimal medium, would be induced by chemicals released during long-term starvation (for more details, see Discussion).

10.1128/mBio.02336-16.4 FIG S3  Regrowth of cells after dilution with supernatant. (A) Design of the experiments. To test whether cells that had stopped growing at ~10^5^ cells/ml could grow again in the supernatant after the first growth cessation (first round), the first-round cultures were diluted 1,000-fold with supernatant obtained from the first-round cultures at 120 h after inoculation (second round). This procedure was performed again (third round) (*n* = 2). (B) Growth curves of cells in the supernatants during the first, second, and third rounds. (C) Final cell concentration for each round. The error bars indicate the standard deviations. Download FIG S3, PDF file, 0.1 MB. Copyright © 2017 Takano et al. 2017 Takano et al. This content is distributed under the terms of the Creative Commons Attribution 4.0 International license.

10.1128/mBio.02336-16.5 FIG S4  Regrowth in the culture after growth cessation in M63 minimal medium supplemented with glucose. (A) Growth curves of fresh cells inoculated into M63 minimal medium supplemented with 0.04% glucose. Viable cells were counted by CFU (*n* = 2). (B) To test whether sufficient nutrients remained for regrowth to the same level as shown in panel A at 24 h, we collected the supernatants from that culture at 24 h. Freshly prepared cells (10^4^ cells/ml) were inoculated into the supernatant, and the growth kinetics of these cells were counted by CFU (*n* = 2). Download FIG S4, PDF file, 0.01 MB. Copyright © 2017 Takano et al. 2017 Takano et al. This content is distributed under the terms of the Creative Commons Attribution 4.0 International license.

Next, we investigated whether the cells controlled absolute growth, death, or both depending on the viable cell density. Figure 3B shows the population growth rate, which is the sum of the absolute growth and death rates. To determine the absolute death and growth rates separately, we measured the dynamics of total cellular particles in the supernatants from cultures of starving cells using flow cytometry (FCM). Counting CFU only provides information on the number of viable cells in the culture, whereas flow cytometry returns the total number of viable and dead cells in the culture by counting all total cellular particles, as shown in Ying et al. (14). The number of total cellular particles counted by flow cytometry (FCM) did not change for at least 6 days after starvation, although 90% of the cells died during this period (see Fig. S5 in the supplemental material). Therefore, the degradation of dead cells is negligible over this short time frame. We can estimate the absolute growth and death rates by calculating [*d*(FCM)/*dt*]/CFU and [*d*(FCM − CFU)/*dt*]/CFU (see Materials and Methods for details).

10.1128/mBio.02336-16.6 FIG S5  (A) Dynamics of total cellular particles estimated by flow cytometry (blue line) and viable cells estimated by CFU (black line) (*n* = 3). The error bars indicate the standard deviations. (B and C) Distributions of total cellular particles detected by flow cytometry. Download FIG S5, PDF file, 0.03 MB. Copyright © 2017 Takano et al. 2017 Takano et al. This content is distributed under the terms of the Creative Commons Attribution 4.0 International license.

Figure 3E shows the transitions in the CFU counts and the numbers of total cellular particles in the supernatant from the day 30 starved culture with an initial density of 10^9^ cells/ml. Into this culture, we inoculated 10^6^ cells/ml of freshly prepared cell culture. At the beginning of the incubation, there was an increase in both the FCM and the CFU count, but both numbers remained constant after a 24-h incubation. We calculated the average growth and death rates from 24 to 96 h to be ~−0.00031 h^−1^ and ~0.0012 h^−1^, respectively. We also performed analysis of variance (ANOVA) for the FCM and CFU time course data from 24 to 96 h but found no significant changes during these time frames (FCM, *n* = 3, *F*_3,8_ = 4.07, *P* = 0.986; CFU, *n* = 3, *F*_3,8_ = 4.07, *P* = 0.059). These results suggest that the numbers of viable cells and dead cells do not change significantly and the absolute growth and death rates are kept very low during the long-term stationary phase. The population growth rate from the culture that was started at 10^2^ cells/ml in the supernatant from the day 30 starved culture with an initial density of 10^9^ cells/ml was ~0.3 h^−1^, and the death rate from 0 to 6 days of the culture in M63 medium without glucose that started at 10^9^ cells/ml was ~0.02 h^−1^ (Fig. 1B). Thus, the cells eventually decreased their absolute growth and death rates to zero during the long-term stationary phase depending on the population density of the viable cells.

These results suggest that the cells can significantly decrease both their absolute growth and death rates to enter into long-term stationary phase depending on the population density of the viable cells. Therefore, the maintenance of viable cells in long-term stationary phase cannot be due simply to the balancing of active growth and death. Instead, attenuation of death and recycling are necessary even though the nutrients are sufficient to maintain a growth rate as high as 0.3 h^−1^, as observed in the experiment whose results are shown in Fig. 3A and B.

In the previous study, the cells did not completely halt growth and death; indeed, the viable cells increased at a rate of <0.02 h^−1^ (13). This rate was much lower than the 0.3 h^−1^ observed in the experiment whose results are shown in Fig. 3A and B. Thus, the attenuation of growth and death observed in the present study may be in agreement with the low death and growth rates reported in previous studies.

### Simple recycling model.

In order to check whether the mechanisms identified in our experiments are sufficient to explain constant survival during starvation, we constructed and analyzed a simple mathematical model. Previously, another mathematical model describing *E. coli* population dynamics during starvation was constructed (18). In contrast to the previous model, we centered our model on the long-term outcomes of starvation and introduced new assumptions based on our experimental results into our model, as follows: (i) the absolute cell death rate depends on the available substrates (Fig. 1B) and the concentration of viable cells (Fig. 3E), and (ii) the absolute cell growth rate depends on both the substrate concentration (Fig. 2A) and the population density in the starved culture (Fig. 3A and E) (for details, see Text S1 in the supplemental material).

**FIG 4:**
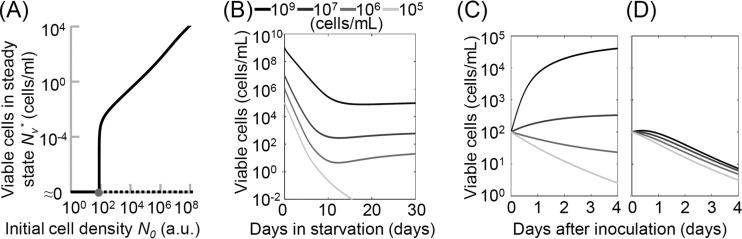
The model constructed based on our assumptions supports the importance of recycling activity and our experimental results. (A) Bifurcation graph of the numbers of viable cells in steady state obtained with our mathematical model. Stable steady states are marked with a solid line, and unstable steady states are marked with a dashed line. (B) Temporal survivability of *E. coli* cells obtained by numerical simulations using the mathematical model. (C and D) Simulated temporal growth kinetics for the cells in the supernatants at day 30 (as shown in Fig. 1C) using the population density-dependent growth (C) or Monod growth [*U*(*S*) = *V*_*m*_*S*/(*K* + *S*)] (D) in the model. To simulate each of the lines, we set the initial substrate concentration to the concentration at day 30 of the same-colored line in panel B. We used the following parameter set for the analysis: *V*_*m*_ = 0.3, *D*_*m*_ = 0.035, *K* = 650, *α* = 120, *β* = 0.001, *γ* = 1, and *r* = 1.0 × 10^−6^.

For simplicity, we describe our experimental system using the following three state variables: viable cells (*N*_*v*_), dead cells (*N*_*d*_), and substrate (*S*) (Fig. 4A). The rates of change of viable cells, dead cells, and substrate are represented by the following model:

$$\begin{array}{l} \frac{dN_{v}}{dt}=G\left(N_{v},S\right)N_{v}-D\left(N_{v},S\right)N_{v}\\ \frac{dN_{d}}{dt}=D\left(N_{v},S\right)N_{v}-\frac{r}{B}N_{d}\\ \frac{dS}{dt}=rN_{d}-cG\left(N_{v},S\right)N_{v} \end{array}$$

where *r* is the substrate secretion rate from dead cells and *G* and *D* are the absolute growth and death rates, respectively, taking the following form (for details, see Text S1 in the supplemental material):

$$\begin{array}{l} G\left(N_{v},S\right)=\frac{1}{c}\cdot \;\frac{V_{m}S}{K+S}\cdot \frac{S}{S+\alpha N_{v}}\\ D\left(N_{v},S\right)=D_{m}\left(\frac{1}{1+\beta S}+\frac{N_{v}}{\gamma S+N_{v}}\right) \end{array}$$

In our model, *c* is the amount of substrate required to yield 1 cell and *B* is the amount of substrate accumulated in each cell. For further details of our mathematical model, see Text S1 in the supplemental material.

### Mathematical analysis of the model is consistent with the importance of recycling during starvation.

Because we wished to focus on stable survival after the cell density stopped decreasing, we studied the steady states of our system. For *c ≈ B* (i.e., the loss of energy through the uptake of metabolizable and releasable substrates is very low), the system has two steady states: a trivial steady state in which the cells cannot survive (i.e., *N*_*v*_ = 0) and a nontrivial steady state in which the cells can survive (i.e., *N*_*v*_* *≠ 0) (Fig. 4A; see also Text S1 in the supplemental material). This condition indicates that a homogeneous culture is able to survive for a long period under starvation conditions through recycling activity alone.

Our model is also able to capture a range of experimental results. First, we show that the initial population density in our model can influence the survival of the bacterial population during starvation, which is in agreement with our experimental observations (Fig. 1A). We examined how the cell viability changed depending on the initial viable cell density. Figure 4A illustrates *N*_v_* (the density of viable cells in steady state) as a function of *N*_0_ and shows the stability of the steady states according to the initial cell density (*N*_0_). When the initial cell density is above a certain threshold, the trivial steady state becomes unstable and a stable nontrivial steady state appears. Thus, there is a threshold of initial cell density below which the cells cannot survive long term, and a high initial cell density enhances cell survivability during starvation.

Figure 4B shows the temporal survival kinetics of viable cells obtained by numerical simulation. This graph is also consistent with our experimental results on the density dependence of the shift between the death and stationary phases.

The model can also explain the growth kinetics in the supernatant of a starved culture, as presented in Fig. 1C and 3C and their legends (Fig. 4C; see also Fig. S6 in the supplemental material). This behavior cannot be predicted when we drop the assumption of the density-dependent growth rate. If we simply use the Monod equation, in which cell growth is regulated only by the substrates, cell regrowth in the supernatant cannot be obtained (Fig. 4D).

10.1128/mBio.02336-16.7 FIG S6  (A) Numerical simulation of the cell growth in the supernatants at day 4 shown in Fig. 3B, using the population density-dependent growth. We set the initial substrate concentration to the concentrations at day 4 shown by the same-colored lines in Fig. 4C. (B) Temporal survival kinetics using various nutrient release rate (*r*) values obtained by numerical simulations of the mathematical model. Under all conditions, the initial cell density was set to 10^9^ cells/ml, and *c* = *B* was applied. (C) Temporal kinetics of the number of viable cells when energy loss is considered (*c *≠ *B*, *B*/*c* = 1.0 × 10^−4^) in the model. (D) Temporal kinetics of survivability using various nutrient release rate (*r*) values when mass conservation is not applied (*c *≠ *B*, *B*/*c* = 1.0 × 10^−4^), obtained by numerical simulations of the mathematical model. Under all conditions, the initial cell density was set to 10^9^ cells/ml. In all analysis of data shown in this figure, we used the following parameter set: *V*_*m*_ = 0.3, *D*_*m*_ = 0.035, *K* = 650, *α* = 120, *β* = 0.001, *γ* = 1, and *r* = 1.0 × 10^−6^. Download FIG S6, PDF file, 0.5 MB. Copyright © 2017 Takano et al. 2017 Takano et al. This content is distributed under the terms of the Creative Commons Attribution 4.0 International license.

Our simple mathematical model demonstrates the hypothesis we formulated based on our experimental results: recycling activity is sufficient to support cell survival during long-term starvation. Moreover, we also find that density dependence of the growth rate is a necessary assumption to explain the observed behavior of the starving culture.

We successfully visualize how the experimentally derived evidences are combined in a rational manner and explain the population dynamics without contradictions between individual evidences.

## DISCUSSION

Our findings showed the mechanism that allows the maintenance of population viability through recycling activity in *E. coli* under starvation conditions more clearly than previous studies. We found a form of the Allee effect (10) in the starved bacterial population: the number of surviving cells in the starved population increased with the increase in the initial population density because the amount of nutrients released into the culture also depended on the population density. This finding supports the previously proposed hypothesis that death and recycling are important for *E. coli* cell survival. In other organisms, cannibalism is also thought to be important for growth or survival under starvation conditions (19), and thus, the recycling of dead cells is perhaps not a mechanism specific to *E. coli*, but a general behavior in bacterial populations. The use of dying cells observed in many other systems has been regarded as an altruistic mechanism (20–23). Perhaps social recycling by *E. coli* might also be supported by altruistic mechanisms (e.g., programed cell death), and this possibility should be explored further.

**FIG 5:**
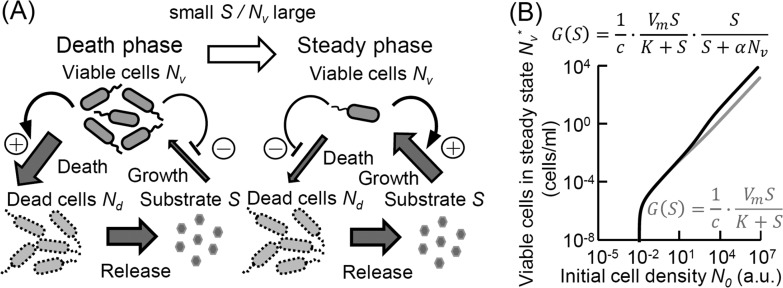
Recycling is restrained until the population reaches a threshold. (A) Schematic diagram of the proposed recycling model. Our experimental results indicate that *E. coli* cells can change their growth rate depending on their density. (B) The number of viable cells in steady state depending on the initial cell density when Monod growth or density-dependent growth was used. We used the following parameter set for the analysis: *V*_*m*_ = 0.3, *D*_*m*_ = 0.03, *K* = 0.1, *α* = 100, *β* = 1, *γ* = 1, and *r* = 1.0 × 10^−5^.

Our quantitative analysis identified another mechanism that enabled the number of surviving cells to remain constant during starvation: the population density-dependent attenuation of growth and death. The viable cells grew when their fraction was smaller than a given threshold and stopped growing when their fraction exceeded the threshold, even if the remaining nutrients were sufficient for further growth in the culture (Fig. 5A). The population density-dependent attenuation in growth and death was not a specific characteristic of the minor mutants in the population but was the standard nature of the wild-type strain in long-term stationary-phase culture (Fig. 3A and B; see Text S1 in the supplemental material for a more detailed discussion).

Is there a merit of controlling the social recycling depending on the population density in long-term stationary-phase culture? Assuming a simple Monod growth model, independent of population density, our mathematical model predicts that faster growth enables cells to survive for a long period even when they are inoculated at a lower population density (see Fig. S7A in the supplemental material). This process prevents extinction when the cells are at a low density. However, fast growth also causes a decrease in the viable cell density at steady state because it converts substrates into biomass faster and results in a lower concentration of substrates in the environment and, therefore, a higher death rate (see Fig. S7). How can the population prevent this trade-off during starvation? One solution may be population density-dependent growth. In this case, cells can increase their growth rate at a low population density and decrease it at a high population density. Figure 5B shows the steady states of the population according to two different models using specific parameter sets. In the first example, the cell growth rate is defined as a Monod function (gray line), and in the second example, the cell growth rate depends on both the substrates and the viable cell density (black line). Figure 5B shows that population density-dependent growth can increase the number of viable cells at steady state without moving the branching point (i.e., the population can still survive at a relatively low population density). Therefore, population density-dependent growth could be beneficial for the survival of a population. This population-density dependent growth might occur specifically in a long-term stationary-phase culture in an artificially constructed environment and might have an unfavorable effect on the cells in nature. However, the results of the numerical simulation indicate that acquiring this sort of regulation might benefit cell survival by increasing the number of survivors under critical conditions like starvation, regardless of whether it is designed adaptation or not.

10.1128/mBio.02336-16.8 FIG S7  Trade-off between shifting the branching point to a higher initial cell density (A) and decreasing the viable cells in the long-term stationary phase (B) when the maximum growth rate is changed. The parameter set used was the same as is listed in the legend to Fig. 5. Download FIG S7, PDF file, 0.1 MB. Copyright © 2017 Takano et al. 2017 Takano et al. This content is distributed under the terms of the Creative Commons Attribution 4.0 International license.

Our results suggest that survival in long-term stationary phase is realized not only by the death of the majority but also by restraint of recycling.

This study provides no evidence to reject the idea that the proposed benefit cannot be adopted in nature. If this population density-dependent growth and death is a reproductive restraint that is observed in a wide range of species (24, 25), how the *E. coli* population evolved this reproductive restraint is also important and should be explored further. As we show in Fig. S7 in the supplemental material, the reproductive restraint observed in this recycling system would increase the number of viable cells in the long-term stationary phase and be beneficial for the population. However, like other cooperative traits, this reproductive restraint might also cause a population to be vulnerable to invasion by cheaters that might not restrain their recycling activity and, thus, might consume all resources available in the environment. This could lead to a classical scenario of the tragedy of the commons, in which greedy individuals cause the collapse of the whole population (26, 27).

One possible mechanism to facilitate cooperative behavior and inhibit invasion by cheaters is the existence of a spatially structured environment (28–31). In a well-mixed liquid culture, it would be difficult to sustain this cooperative trait because both cells and resources from dead cells can diffuse freely throughout the environment. In this case, potential scavengers that lose the density-dependent regulation can easily dominate the population. However, if the habitat is spatially structured (e.g., when bacteria form colonies or biofilms, as in the case of infections of the human body), density-dependent growth would be sustained. In this case, nutrients from dead cells would diffuse slowly to the environment, and most of the resources would remain within the neighborhood of the “cooperative cells” (i.e., those which exhibit the density-dependent growth). In this case, the patches that contain cheaters would have little chance to maintain a higher survival rate than those consisting of only cooperators (cells that exhibit density-dependent growth), as in the scenario often discussed in group selection theory (21, 30, 32, 33).

Importantly, in this evolutionary scenario, conditions favorable for the cooperators might be restricted or sparse, since patches with the high cell density required for the density-dependent growth are also likely to contain cheaters.

Our results and those of previous studies showed that nutrients from dead cells can enhance survivability in starvation, but how these nutrients are released in a form that can be utilized by viable cells should be studied further. Cell death seems to cause degradation of cellular components and diffusion of degraded particles at any time, but this hypothesis needs further investigation. It has been suggested that degradation of ribosomes supplies carbon and energy sources, such as ribose and amino acids, to *E. coli* cells upon the shift to starvation (34–37). This process would be important for accumulating internal resources for synthesizing the proteins needed to adapt to nutrient deficiency (38). This process may also be important for recycling activity in the long-term stationary phase. If there was only natural decay of macromolecular proteins after cells die, it would be harder for the survivors to take up and metabolize these resources. On the contrary, if cells actively degraded ribosomes and produced compounds that are easily metabolized before and/or after they die, this would also be beneficial for scavengers. Some GASP (growth advantage in stationary phase) mutants have been shown to have an increased ability to catabolize some amino acids (6–9); it is plausible that amino acids are an important resource for survivors during long-term starvation. The importance of protein degradation not only for individual intracellular activity but also for group benefit should be explored further.

How *E. coli* cells perform this density-dependent recycling during starvation should be studied in more detail. Since the population density-dependent growth is not observed in any freshly prepared medium, it is expected to be induced by specific compounds in the starved-culture medium. Could it be mediated by the secretion of signaling molecules like quorum-sensing molecules? Previous studies showed that a small metabolite (homoserine lactone [HSL]) that is structurally related to quorum-sensing signals (acylated HSLs) affected the levels of the sigma factor RpoS in *E. coli* cells (39, 40). The amount of RpoS greatly affected *E. coli* population viability during starvation and cell growth (12, 41–43). Therefore, starving *E. coli* cells may sense the population density by the quorum-sensing mechanism and change their growth and death rates accordingly. However, if starving *E. coli* cells control their growth and death rates by quorum sensing, small molecules that decrease the cell growth and death rates at a high cell density should accumulate in the culture. In this case, there would be little opportunity for growth in the supernatant of the starved culture. However, in our experiments, cells grew in the supernatants of the starved cultures (Fig. 3A to D). Thus, a short half-life of such diffusible molecules would be necessary to sense population density or another mechanism would need to be at work (e.g., cell-cell contact). However, it would be implausible, or at least atypical, that physical interactions would enable the cells to sense their population density, because density-dependent growth arrest was observed at a very low cell density (10^5^ cells/ml). Thus, diffusible molecules that have high degradation rates would be a more plausible mechanism for density-dependent growth.

Understanding the mechanism of social recycling at a molecular level may provide us with a unique opportunity to manipulate bacterial population behavior in industrial or medical environments.

## MATERIALS AND METHODS

### Strains and culture conditions.

The reduced-genome *E. coli* strain MDS42 was purchased from Scarab Genomics (Madison, WI). The bacterial cells were cultured in M63 minimal medium [62 mM K_2_HPO_4_, 39 mM KH_2_PO_4_, 15 mM (NH_4_)_2_SO_4_, 2 μM FeSO_4_⋅7H_2_O, and 203 μM MgSO_4_⋅7H_2_O] with 22 mM glucose at 37°C with shaking at 160 rpm in a Br-21FP air incubator (Taitec, Saitama, Japan). For the glucose starvation assay, *E. coli* cells were grown in M63 medium with glucose at a density of approximately 2 × 10^9^ cells/ml and washed three times with M63 minimal medium without glucose. Then, samples were diluted to the initial population density indicated for each experiment. For competition experiments and counting cellular particles, the MDS42*ΔgalK*::*gfp-*Km^r^ strain was used (44). For validation, we performed each experiment on different days using different inocula. We also used supernatant obtained from a different starvation culture for each experiment.

### Estimation of cell viability.

We estimated cell viability by counting CFU after glucose starvation. Cells incubated under each condition were inoculated onto M63 (0.4% glucose) agar (1.5%) plates. The plates were incubated at 37°C for more than 40 h. After incubation, the colonies were manually counted (*n* = 2). The number of viable cells was considered zero if there were no colonies on the plate inoculated with 1 ml of the culture.

### Preparation of supernatants.

The cultures of glucose-starved *E. coli* cells were centrifuged at 10,000 rpm for 3 min at room temperature. The supernatants were filtered with a 0.22-μm filter (EMD Millipore, Billerica, MA) and stored at 4°C.

### Estimation of the numbers of total cellular particles.

The total-cellular-particle concentrations were determined using a flow cytometer (FACSCanto II; Becton, Dickson and Company, Franklin Lakes, NJ). The following photomultiplier tube (PMT) voltage settings were applied: forward scattering (FSC), 560 V, and side scattering (SSC), 400 V. For calibration, 3-μm Fluoresbrite yellow green (YG) microsphere beads (Polysciences, Warminster, PA, United States) were used. Using a fluorescent strain (MDS42*ΔgalK*::*gfp-*Km^r^), we first validated the appropriate region of the plots to discriminate cellular particles from other particles by size (FSC) and fluorescence signal (fluorescein isothiocyanate [FITC]). Then, we set the region where all cellular particles recognized by the FSC-FITC plot could be discriminated from other particles in the cell size plot (FSC-SSC plot). We defined the particles included in that region as cellular particles. Then, we estimated the numbers of cellular particles of the nonfluorescent strains by size. We also measured the particle counts of the bead-only culture and verified that few particles were counted in that region. The flow cytometry datasets for FSC and SSC, which measured the relative cell size and morphology, respectively (45), were analyzed using custom MATLAB (MathWorks, Natick, MA) scripts. Because the decrease in dead cells as particles is negligible over a short period, the following equations were applied:

$$\begin{array}{l} G=\frac{1}{N_{v}}\cdot \frac{d\left(N_{v}+N_{d}\right)}{dt}=\frac{1}{\text{CFU}}\cdot \frac{d\left(\text{FCM}\right)}{dt}\\ D=\frac{1}{N_{v}}\cdot \frac{dN_{d}}{dt}=\frac{1}{\text{CFU}}\cdot \frac{d\left(\text{FCM}-\text{CFU}\right)}{dt} \end{array}$$

### Estimation of the population growth rate in the supernatant.

The population growth rate (the sum of the absolute growth and death rates, *μ*) was estimated by fitting the experimental results to the following logistic equation with the lsqcurvefit function (MATLAB; MathWorks, Natick, MA):

$$N_{v}=\frac{K}{1+\left(\frac{K-N_{0}}{N_{0}}\right)e^{-\mu t}}$$

### Computation of the bifurcation graph and survival kinetics.

To obtain survival curves by numerical simulation, we implemented the mathematical model as a system of ordinary differential equations (ODE) and performed the numerical calculation using the *ode15s* function in MATLAB. The computation of steady states and the bifurcation graphing of the ODE system were performed by using Matcont (version 2.4) (46).

### Measurement of the Nal^r^ mutation rate.

The rate of mutation for resistance to nalidixic acid (Nal^r^) was estimated using a fluctuation test (47). Based on a previous report (48), the strains were inoculated into 5 ml of medium and cultured at 37°C for 22 h. The cultures were centrifuged at 7,500 rpm for 5 min at room temperature, and the pellets were plated on LB agar plates supplemented with 80 μg/ml of nalidixic acid. Then, these plates were incubated at 37°C for more than 40 h, and the number of plates containing Nal^r^ colonies was counted manually. We estimated the mutation rate using 20 cultures. The mutation rate (substitution/replication*base) was calculated by estimating the fraction of cultures containing no mutations among 20 cultures (*p*_0_ method) as previously described (47). The total genome size of MDS42 was ~4.0 × 10^6^ bases, and the genomic mutation rate was calculated by multiplying the mutation rate per base by the genome size.
