# Supplementary material for: Density-Dependent Recycling Promotes the Long-Term Survival of Bacterial Populations during Periods of Starvation
Source: mBio. 2017 Feb 7;8(1):e02336-16. doi: 10.1128/mBio.02336-16 (PMC5296608; doi:10.1128/mBio.02336-16)
Supplement: FIG S1 [file mbo001173171sf1.pdf]

Fig. S1

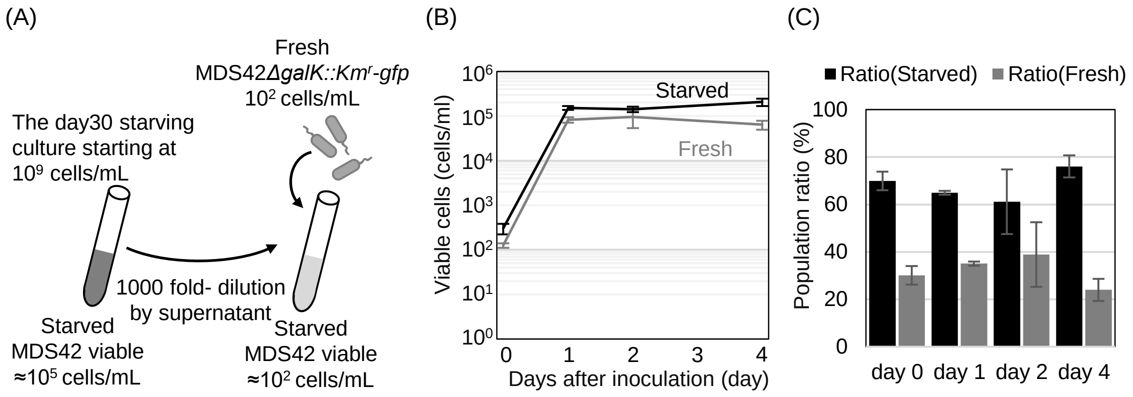

Figure S1. Competition experiments between cells starved for 30 days and freshly prepared cells. (A) Design of the experiments. To test whether evolution or selection during 30 days' starvation enhances the ability of viable cells to grow using nutrients from dead cells, we diluted MDS42 day 30 cell cultures (10^9 cells/mL) with supernatant from the same cultures, and mixed the diluted cultures with freshly prepared cell cultures (MDS42Δ*galK::gfp-Km*^{r}). (B) Growth curves of starved and fresh cells in the mixed population in the supernatant estimated by CFUs. To estimate the viability of both strains, cell cultures were plated on M63 agar plate with or without Kanamycin at 40 μg/mL. The viability of fresh cells was estimated from a viable count on M63 agar plate with Kanamycin, and that of starved cells was estimated by subtracting a viable count on M63 agar plate with Kanamycin from that on M63 agar plate without Kanamycin. (C) Transition of population ratio in mixed cultures from day 0 to 4. No significant changes were detected in this time course (ANOVA, n=3, $F_{3,8}$ = 4.07, P = 0.29). The error bars indicate the standard deviations.
