# Supplementary material for: Density-Dependent Recycling Promotes the Long-Term Survival of Bacterial Populations during Periods of Starvation
Source: mBio. 2017 Feb 7;8(1):e02336-16. doi: 10.1128/mBio.02336-16 (PMC5296608; doi:10.1128/mBio.02336-16)
Supplement: TEXT S1 [file mbo001173171s1.pdf]

Supplementary Text

Rationale for the survival strategy without *de novo* mutations. Our results proposed a rationale for a survival strategy without de novo mutations. Because the mutation rate of the strain used in this study is very small (~1.0×10^{-3} substitutions/genome · replication), the population in our experiments is considered nearly genetically homogeneous. Thus, the behavior of the majority (>99% of the population) during starvation (i.e., death and release of substrates) might not result in genetic diversification. Indeed, it is possible that a small proportion of the survivors during long-term starvation acquires mutations to survive because the emergence of 1.0×10^{-3} % mutants is expected on average (e.g., 10^6 cells/mL mutants in a 10^9 cells/mL population); however, this possibility may also not be observed. Because the emergence of mutations in a population is a random and rare event (1), there is little possibility to observe reproducible survival kinetics during starvation in experiments (Figure 1a). Moreover, our experimental results support the hypothesis that a small minority of survivors would not need to acquire any mutations. Freshly prepared cell cultures from the wild-type strain showed the maintenance of the viable cells in the supernatant from starved cultures at the same level as observed during long-term stationary phase.

Therefore, it is plausible that survival in long-term stationary phase does not necessarily require specific mutations or genetic diversification in the population.

The model description for bacterial survival in starvation using three variables. To verify whether the mechanisms identified in our experiments (i.e., death and recycling coupled with restraint in recycling activity) sufficient to explain constant survival during starvation, we constructed a simple mathematical model focused on the following assumptions rooted in the experimental findings. i) Viable cells are able to

uptake nutrients released from the dead cells. For simplicity, we assume that the nutrients are released at a constant rate *r;* ii) the culture is homogeneous; therefore, we do not take into account any genetic variations; iii) the absolute death rate depends on the available substrate (Figure 1b: there is no growth, but the kinetics of the population death rate change depending on the released substrate concentration) and the concentration of viable cells (Figure 3d: the death rate during the long-term stationary phase is smaller than the death rate during death phase even though the substrate concentration does not change); and iv) the absolute cell growth rate depends on both the substrate concentration (Figure 2a) and the population density (Figure 3a and b) in the starved culture.

The dynamics of the bacterial survival rate during starvation with the effect of releasing and utilizing nutrients from dead cells is governed by three equations (1)−(3). There are three variables: concentration of a substrate in a culture ($S[\mu g \cdot ml^{-1}]$), the number of viable cells (N_v $[cells \cdot ml^{-1}]$), the number of dead cells (N_d $[cells \cdot ml^{-1}]$). These variables can be described as functions of $t[h]$. Although there is variation in the available carbon sources in the real world, for simplicity we do not differentiate between them and instead consider all of the carbon sources as a single energy source (S).

$G(N_v, S)[h^{-1}]$ is the growth rate and $U(N_v, S)[\mu g \cdot h^{-1}]$ is the uptake rate of the substrate. Then, the growth rate $G(N_v, S)$ is given by $G(N_v, S) = U(N_v, S)/c$, where $c[\mu g \cdot cells^{-1}]$ is the amount of substrate required to yield one cell. $D(N_v, S)[h^{-1}]$ is the death rate of the viable cells;

$r[\mu g \cdot h^{-1}]$ is the rate of releasing of the substrate from the dead cells and $B[\mu g \cdot cells^{-1}]$ is the amount of substrate accumulated in each cell. For simplification, r and B are constant and are not variable in the population.

To obtain mass conservation we assume that $c \cong B$ (i.e., the loss of energy through absorbing, metabolizing and releasing substrates is very low). When this assumption is not applied in this system, comparatively constant survival for a month was observed in numerical simulations. For example, we additionally show survival kinetics when using B = 1/10000*c (Fig. S6C). In this condition, attenuation of the decrease in cell viability and comparatively constant survival for 30 days were observed. Thus B > 1/10000*c can be considered as a small energy loss in this time-scale. However, in order to apply the mathematical analysis of the steady states (Fig 4a), we must assume $c \cong B$.

$$\frac{dN_v}{dt} = \frac{U(N_v, S)}{c} N_v - D(N_v, S) N_v \tag{6.1}$$

$$\frac{dN_d}{dt} = D(N_v, S) N_v - \frac{r}{c} N_d \tag{6.2}$$

$$\frac{dS}{dt} = rN_d - U(N_v, S) N_v \tag{6.3}$$

The uptake rate $U(N_v, S)$ is a function of the viable cells and substrates. $V_{max}[\mu g \cdot h^{-1}]$ and $K[\mu g]$ are defined as the maximum uptake rate and half saturation constant, respectively.

$$U(N_v, S) = V_{max} \frac{S}{K + S} \cdot \frac{S}{S + \alpha N_v} \tag{2}$$

$D(N_v, S)$ is a function of N_v and S. The following equation is given.

$$D(N_v, S) = D_{max} \left(\frac{1}{1 + \beta S} + \frac{N_v}{\gamma S + N_v} \right) \tag{3}$$

Computation of steady states. The trivial steady state from (1) is

$$N_v{}^* = 0 \tag{4}$$

if $N_v{}^* = 0$, $N_d{}^*$ is calculated from (2).

$$N_d{}^* = 0 \tag{5}$$

In this case, S^* can take any value in this steady state. The trivial steady state ($\boldsymbol{N}^{*0}$) from (1)-(3) is given as follows. In this case, S^0 can take any value, depending on the initial conditions. .

$$\boldsymbol{N}^{*0} = \begin{pmatrix} N_v{}^0 \\ N_v{}^0 \\ S^0 \end{pmatrix} = \begin{pmatrix} 0 \\ 0 \\ S^0 \end{pmatrix} \tag{6}$$

Next, consider the non-trivial steady state ($\boldsymbol{N}^{*1}$) (i.e., the number of viable cells in steady state is not zero: $N_v{}^* \neq 0$) from (1)-(3). If $N_v{}^* \neq 0,$ the following equation has to be satisfied.

$$\frac{U(N_v{}^*, S^*)}{c} = D(N_v{}^*, S^*) \tag{7}$$

Then, $N_d{}^*$ is computed from (2).

$$D(N_v{}^*, S^*)N_v{}^* - \frac{r}{c}N_d{}^* = 0 \tag{8}$$

$$N_d{}^* = \frac{cD(N_v{}^*, S^*)}{r}N_v{}^* \tag{9}$$

$\boldsymbol{N}^{*1}$ is described as follows. $N_v{}^\alpha$ and S^α are the density of viable cells and the concentration of substrate satisfying (9), respectively.

$$\boldsymbol{N}^{*1} = \begin{pmatrix} N_v{}^1 \\ N_d{}^1 \\ S^1 \end{pmatrix} = \begin{pmatrix} N_v{}^\alpha \\ \dfrac{cD(N_v{}^\alpha, S^\alpha)}{r}N_v{}^\alpha \\ S^\alpha \end{pmatrix} \tag{10}$$

Next, take into account an initial condition to compute the steady state. Here, I assume that there are no substrates and dead cells at the beginning of starvation in the culture. Therefore, the initial value of each parameter $\boldsymbol{N}^I$ is given as follows.

$$\boldsymbol{N}^I = \begin{pmatrix} N_v{}^I \\ N_d{}^I \\ S^I \end{pmatrix} = \begin{pmatrix} N_0 \\ 0 \\ 0 \end{pmatrix} \tag{11}$$

N_0 is the initial density of viable cells in the culture. Then, the following mass conservation equation is applicable for every t.

$$cN_0 = cN_v + cN_d + S \tag{12}$$

in the case of N_v-zero steady state, ${N_v}^0 = {N_d}^0 = 0$. Then, S^0 is

$$S^0 = cN_0. \tag{13}$$

Therefore $\boldsymbol{N}^{*0}$ is modified as

$$\boldsymbol{N}^{*0} = \begin{pmatrix} {N_v}^0 \\ {N_d}^0 \\ S^0 \end{pmatrix} = \begin{pmatrix} 0 \\ 0 \\ cN_0 \end{pmatrix} \tag{14}$$

Next, consider the non-trivial steady state. From (11), the conservation equation (14) is modified as

$$N_0 = {N_v}^* + \frac{cD({N_v}^*, S^*)}{r} {N_v}^* + \frac{S^*}{c} \tag{15}$$

and further modified as

$${N_v}^* = \frac{r}{r + cD({N_v}^*, S^*)} \left(N_0 - \frac{S^*}{c} \right) \tag{16}$$

At least one pair of ${N_v}^*$ and S^* have to satisfy equation (9) and (18). If no ${N_v}^*$and S^*satisfy both the two equations, then the non-trivial steady state does not exist.${N_v}^*$ in (18) is substituted for ${N_d}^*$ in (11). ${N_v}^\beta$ and S^β are the density of viable cells and the concentration of substrate satisfying both (9) and (18), respectively.

$${N_d}^* = \frac{cD\left({N_v}^\beta, S^\beta\right)}{r + cD\left({N_v}^\beta, S^\beta\right)} \left(N_0 - \frac{S^\beta}{c} \right) \tag{17}$$

Using these results, $\boldsymbol{N}^{*1}$ can be described as follows.

$$\boldsymbol{N}^{*1} = \begin{pmatrix} {N_v}^1 \\ {N_d}^1 \\ S^1 \end{pmatrix} = \begin{pmatrix} {N_v}^\beta \\ \dfrac{cD\left({N_v}^\beta, S^\beta\right)}{r + cD\left({N_v}^\beta, S^\beta\right)} \left(N_0 - \dfrac{S^\beta}{c} \right) \\ S^\beta \end{pmatrix} \qquad (cN_0 > S^\beta) \tag{18}$$

Analyzing survival kinetics when changing parameters *r*, *B* and *c*.

We checked the effect of the parameters r, B and c on the survival kinetics. Because a range of these parameters was not estimated in our experiments, we changed the values of these parameters and checked how the population dynamics are affected by them.

At first, we observed survival kinetics when applying various r, the rate of releasing the substrates, to the system. Fig. S6B shows temporal kinetics of the number of viable cells for 30 days. r has effects on both death rate and the number of viable cells in long-term stationary phase. If the releasing rate becomes lower, survivability decreases faster and the viable cell density in steady state reduces. Therefore, fast release of the substrates would enhance survivability when the energy-loss is negligible ($c \cong B$).

We also checked survival kinetics in numerical simulations when mass conservation is not applied in this system ($c \neq B$). For example, we additionally show survival kinetics of numerical simulation when using B = 1/10000*c (Fig. S6C). We can assume that B > 1/10000*c because starvation culture of 10^9 cells/mL can release nutrients sufficient to grow 10^5 cells/mL (Fig. 1c). Even in this scenario, attenuation of the decrease in cell viability and comparatively constant survival for 30 days were observed in the numerical simulation. Thus B = 1/10000*c can be considered as a small energy loss in this time-scale.

Next, we studied how the releasing rate has an influence on survivability when mass conservation is not applied in this system. Fig. S6D shows temporal survival kinetics obtained by numerical simulation when B = 1/10000*c, and there is not a great difference even though r is changed. This graph demonstrates that fast release rate may not enhance the survivability in this system, if there is a large energy loss. This is because a quick release of the substrates to the environment would cause a loss of a

huge amount of substrates, and not necessarily increase the substrate concentration in the environment.

Reference

1. **Luria SE, Delbruck M.** 1943. Mutations of Bacteria from Virus Sensitivity to Virus Resistance. Genetics **28:**491-511.
