# Supplementary material for: Density-Dependent Recycling Promotes the Long-Term Survival of Bacterial Populations during Periods of Starvation
Source: mBio. 2017 Feb 7;8(1):e02336-16. doi: 10.1128/mBio.02336-16 (PMC5296608; doi:10.1128/mBio.02336-16)
Supplement: FIG S2 [file mbo001173171sf2.pdf]

Fig. S2

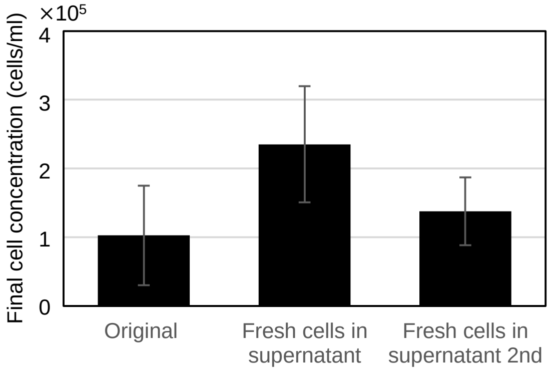

Figure S2. Growth cessation before exhaustion of nutrients in a supernatant. (a) Average viable cell concentrations in original 10^9 cells/mL starved cultures at day 30 (see Fig. 1A, n = 8) and in populations regrown in the supernatant at 96 h after inoculation (first (n = 3) and second rounds (n = 6)) (as shown in Fig. 3B and C). The experimental design is as shown in Figs. 1A, 3B and C. To clarify differences among those samples, we have plotted them against a linear scale. The error bars indicate the standard deviations.
