# Supplementary material for: Density-Dependent Recycling Promotes the Long-Term Survival of Bacterial Populations during Periods of Starvation
Source: mBio. 2017 Feb 7;8(1):e02336-16. doi: 10.1128/mBio.02336-16 (PMC5296608; doi:10.1128/mBio.02336-16)
Supplement: FIG S3 [file mbo001173171sf3.pdf]

Fig. S3

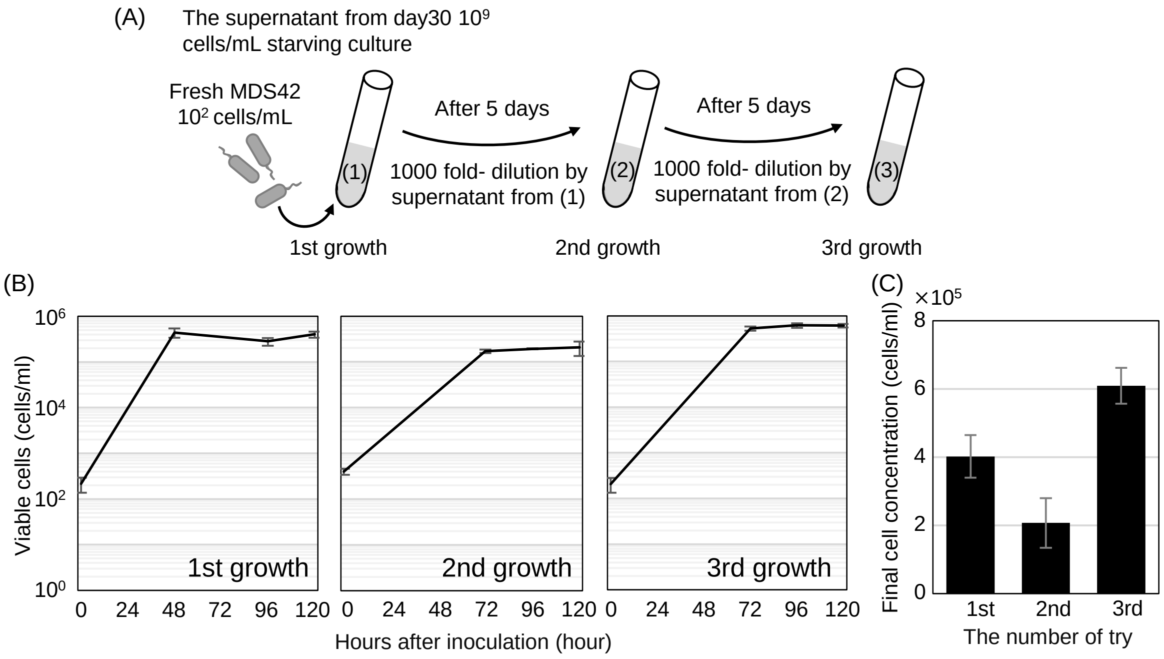

Figure S3. Regrowth of cells after dilution with supernatant. (A) The design of the experiments. To test whether cells that had stopped growing at ~10^5 cells/mL could grow again in the supernatant after the first growth cessation (first round), the first-round cultures were diluted 1000-fold with supernatant obtained from the first-round cultures at 120 h after inoculation (second round). This procedure was performed again (third round). (n = 2). (B) Growth curves of cells during the first, second, third rounds in the supernatant. (C) Final cell concentration for each round. The error bars indicate the standard deviations.
