# Supplementary material for: Density-Dependent Recycling Promotes the Long-Term Survival of Bacterial Populations during Periods of Starvation
Source: mBio. 2017 Feb 7;8(1):e02336-16. doi: 10.1128/mBio.02336-16 (PMC5296608; doi:10.1128/mBio.02336-16)
Supplement: FIG S4 [file mbo001173171sf4.pdf]

Fig. S4

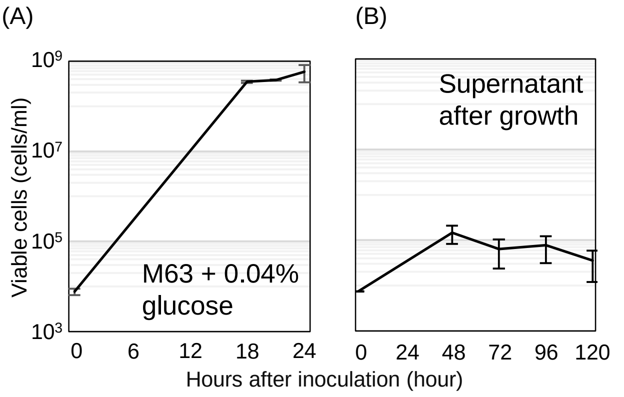

Figure S4. Regrowth in the culture after growth cessation in M63 minimal medium supplemented with glucose. (a) Growth curves of fresh cells inoculated in M63 minimal medium supplemented with 0.04% glucose. Viable cells were counted by CFUs (n = 2). (b) To test whether sufficient nutrients remained to regrow to the same level as those at 24 h in the left panel (A), we collected the supernatants at 24 h from (A). Freshly prepared cells (10^4 cells/mL) were inoculated into the supernatant and the growth kinetics of these cells were counted by CFUs (n = 2).
