# Supplementary material for: Density-Dependent Recycling Promotes the Long-Term Survival of Bacterial Populations during Periods of Starvation
Source: mBio. 2017 Feb 7;8(1):e02336-16. doi: 10.1128/mBio.02336-16 (PMC5296608; doi:10.1128/mBio.02336-16)
Supplement: FIG S5 [file mbo001173171sf5.pdf]

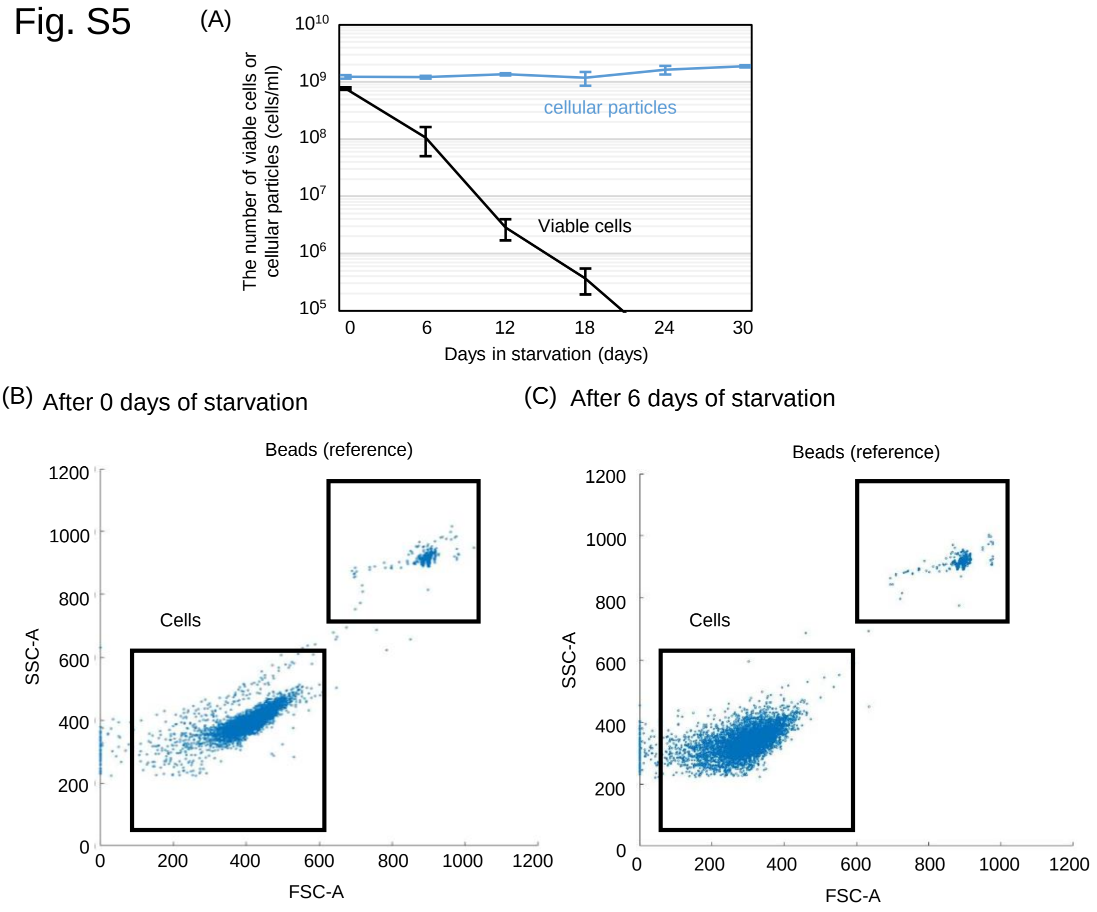

Figure S5. (a) The dynamics of total cellular particles estimated by flow cytometry (blue line) and viable cells estimated by CFUs (black line) (n = 3). The error bar indicates the standard deviations. (b) (c) Distributions of total cellular particles detected by flow cytometry.
