# Supplementary material for: Density-Dependent Recycling Promotes the Long-Term Survival of Bacterial Populations during Periods of Starvation
Source: mBio. 2017 Feb 7;8(1):e02336-16. doi: 10.1128/mBio.02336-16 (PMC5296608; doi:10.1128/mBio.02336-16)
Supplement: FIG S6 [file mbo001173171sf6.pdf]

Fig. S6

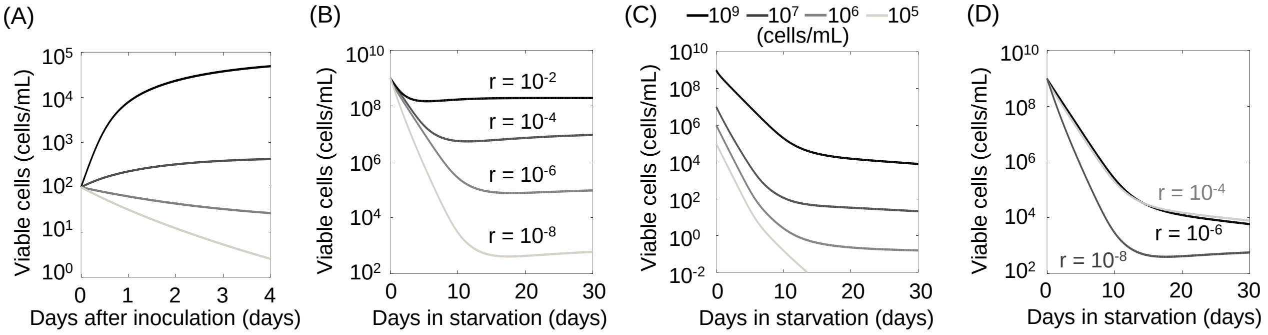

Figure S6. (a) Numerical simulation of the cell growth in the supernatant at day 4 from the Fig. 3B, using the population density-dependent growth. We set the initial substrate concentration to the concentration at day 4 of same colored line in Fig. 4C. (b) Temporal survival kinetics using various releasing rate *r* values obtained by numerical simulations of the mathematical model. In all conditions, initial cell density were set to 10^9 cells/mL, and c = B were applied. (c) Temporal kinetics of the number of viable cells when energy loss is considered (c ≠ B, B / c = 1.0 × 10^{-4}) in the model. (d) Temporal kinetics of survivability using various releasing rate *r* values when mass conservation is not applied (c ≠ B, B / c = 1.0 × 10^{-4}) obtained by numerical simulations of the mathematical model. In all conditions, initial cell density were set to 10^9 cells/mL. In all analysis in this figure, we used the following parameter sets for the analysis: $V_m = 0.3$, $D_m = 0.035$, K =650, $\alpha = 120$, $\beta = 0.001$, $\gamma = 1$, and $r = 1.0 \times 10^{-6}$.
