# Supplementary material for: Density-Dependent Recycling Promotes the Long-Term Survival of Bacterial Populations during Periods of Starvation
Source: mBio. 2017 Feb 7;8(1):e02336-16. doi: 10.1128/mBio.02336-16 (PMC5296608; doi:10.1128/mBio.02336-16)
Supplement: FIG S7 [file mbo001173171sf7.pdf]

Fig. S7

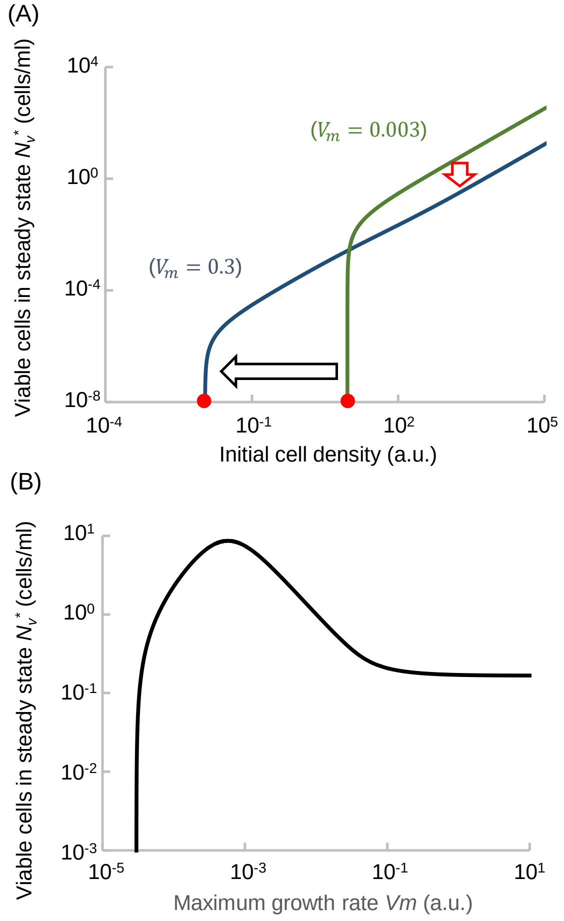

Figure S7. Trade-off between shifting the branching point to a higher initial cell density (a) and decreasing the viable cells in the long-term stationary phase (b) when the maximum growth rate is changed. The used parameter sets are same as those in Fig. 5.
